# Effects of a wide range of dietary nicotinamide riboside (NR) concentrations on metabolic flexibility and white adipose tissue (WAT) of mice fed a mildly obesogenic diet

**DOI:** 10.1002/mnfr.201600878

**Published:** 2017-04-13

**Authors:** Wenbiao Shi, Maria A. Hegeman, Dorien A.M. van Dartel, Jing Tang, Manuel Suarez, Hans Swarts, Bart van der Hee, Lluis Arola, Jaap Keijer

**Affiliations:** ^1^ Human and Animal Physiology Wageningen University Wageningen The Netherlands; ^2^ Department of Biochemistry and Biotechnology University Rovira Virgili Tarragona Spain; ^3^ Nutrition and Health Research Group Technological Center of Nutrition and Health (CTNS) Reus Spain; ^4^ Institute of Animal Sciences Chinese Academy of Agricultural Sciences (CAAS) Beijing China

**Keywords:** Metabolic flexibility, NAD^+^, Niacin, Nicotinamide riboside, Vitamin B3

## Abstract

**Scope:**

Metabolic flexibility is the ability to switch metabolism between carbohydrate oxidation (CHO) and fatty acid oxidation (FAO) and is a biomarker for metabolic health. The effect on metabolic health of nicotinamide riboside (NR) as an exclusive source of vitamin B3 is unknown and is examined here for a wide range of NR.

**Design and methods:**

Nine‐week‐old male C57BL/6JRcc mice received a semi‐purified mildly obesogenic (40 en% fat) diet containing 0.14% L‐tryptophan and either 5, 15, 30, 180, or 900 mg NR per kg diet for 15 weeks. Body composition and metabolic parameters were analyzed. Metabolic flexibility was measured using indirect calorimetry. Gene expression in epididymal white adipose tissue (eWAT) was measured using qRT‐PCR .

**Results:**

The maximum delta respiratory exchange ratio when switching from CHO to FAO (maxΔRER_CHO1→FAO_) and when switching from FAO to CHO (maxΔRER_FAO→CHO2_) were largest in 30 mg NR per kg diet (30NR). In eWAT, the gene expression of *Pparγ*, a master regulator of adipogenesis, and of *Sod2* and *Prdx3*, two antioxidant genes, were significantly upregulated in 30NR compared to 5NR.

**Conclusion:**

30NR is most beneficial for metabolic health, in terms of metabolic flexibility and eWAT gene expression, of mice on an obesogenic diet.

**Abbreviations**
2PY: 2‐pyridone
BW: Body weight
CHO: Carbohydrate oxidation
CLS: Crown‐like structure
EE: Energy expenditure
eWAT: epididymal white adipose tissue
FAO: Fat acid oxidation
HFD: High fat diet
LFD: Low fat diet
MeNam: N‐methylnicotinamide
mtDNA: mitochondrial DNA
NA: Nicotinic acid
NAD+: Nicotinamide adenine dinucleotide
Nam: Nicotinamide
NaMN: Nicotinic acid mononucleotide
nDNA: nuclear DNA
NEFA: Non‐esterified fatty acids
NMN: Nicotinamide mononucleotide
NR: Nicotinamide riboside
RER: Respiratory exchange ratio
TG: Triglycerides
Trp: Tryptophan
WAT: white adipose tissue.

## 1 Introduction

Nicotinamide adenine dinucleotide (NAD^+^) is essential to maintain cellular redox state and basal energy metabolism. Beyond its role as a reusable coenzyme, NAD^+^ is also permanently degraded by various signalling enzymes, including sirtuins 1. Although the degradation product, nicotinamide (Nam), can be recycled for NAD^+^ generation, maintenance of NAD^+^ levels is dependent on exogenous supplementation with vitamin B3, from which NAD^+^ can be synthesized. Indeed plasma NAD^+^ levels decrease when humans or rodents are exposed to a vitamin B3‐deficient diet 2, 3, 4. Severe vitamin B3 deficiency causes pellagra, a disease characterized by dermatitis, diarrhea, and dementia, ultimately resulting in death 5. Even though pellagra has become rare in developed countries, it remains endemic in underdeveloped countries 6.

While severe vitamin B3 deficiency results in disease, hardly any information is available on physiological consequences of marginal vitamin B3 levels. This is particularly relevant because it was shown that obesogenic diets reduce NAD^+^ levels in several tissues 7, 8, 9, 10.Surprisingly, requirements for dietary vitamin B3 are still not well‐established for rodents, especially mice 11, 12. The National Research Council suggested the vitamin B3 requirement in rats to be 15 mg/kg diet, based on three scientific publications in the 1940s. However, these studies did not use purified diets and only used growth rate as a read‐out parameter 13, 14, 15. More importantly, requirements were based on studies with rats as no direct data were available for mice. Estimation of vitamin B3 requirements is not straightforward, since NAD^+^ can also be synthesized de novo from the essential amino acid tryptophan (Trp) 16. Dietary Trp may thus rescue vitamin B3 deficiency. Indeed, rodents can maintain optimal growth and tissue NAD^+^ levels when supplied with a vitamin B3‐free diet containing 0.23% L‐Trp 17, 18. It is, however, not possible to simply use Trp‐deficient diets to establish vitamin B3 requirements, because Trp is essential for serotonin synthesis and protein synthesis 19. The lack of data on vitamin B3 requirement thresholds precludes research into the molecular, metabolic and physiological consequences of marginal status of various forms of vitamin B3.

Nicotinic acid (NA) and Nam are the classic forms of vitamin B3, present in the diet. NA and Nam can be converted to NAD^+^ via the Preiss‐Handler and the salvage pathway, respectively 20. Nicotinamide riboside (NR) is another source of vitamin B3 that naturally exists in cow's milk and yeast‐containing food products. NR can be metabolized into NAD^+^ directly via the Nrk pathway 21. Recent studies have shown that supplementation of NR at pharmacological levels can provide physiological or metabolic benefits by boosting NAD^+^ levels 8, 22, 23, 24, 25, 26. No side effects of NR have been reported, in contrast to high dose treatment with NA, that may cause skin flushing, or Nam, that may lead to liver damage 27. Therefore, NR has been proposed as the vitamin B3 of choice. Despite the benefits from pharmacological NR supplementation 8, 22, 24, 26, it would be necessary to re‐evaluate the NR dose, as it appears strikingly high (400 mg NR/kg body weight/day) compared to most commercially available supplements (60–500 mg/person/day) 28.

The metabolic health effects of NR as an exclusive source of vitamin B3 at nutritional relevant levels are unknown. Recently, the ability to rapidly switch metabolism between carbohydrate oxidation and fatty acid oxidation, so‐called metabolic flexibility, has been recognized as a sensitive biomarker for metabolic health in nutritional interventions 29, 30, 31, 32. Metabolic flexibility can be assessed by analysing the respiratory exchange ratio (RER) during a fast‐refeeding challenge in non‐invasive indirect calorimetry. To examine the effects of nutritional relevant levels of NR on metabolic health, we performed a dose‐response dietary intervention study using a wide range of NR, from 5 to 900 mg NR per kg of a defined semi‐purified obesogenic diet, combined with a metabolic flexibility measurement. We used a mildly obesogenic diet (40 en% fat) to mimic a Western diet. To limit NAD^+^ biosynthesis from Trp, we implemented a low level of Trp (0.14%). This Trp level is still sufficient for normal growth in mice 33. Since white adipose tissue (WAT) may play a crucial role in metabolic flexibility at the whole body level 34, 35, 36, we focused on metabolic flexibility and WAT function.

## 2 Materials and methods

### 2.1 Dietary intervention

The animal experiment was approved by the Animal Welfare Committee of Wageningen University, Wageningen, The Netherlands (DEC2014029). Nine‐week‐old male C57BL/6JRcc mice (Envigo, Horst, The Netherlands) were individually housed (12 h light–dark cycle, 23 ± 1°C, 55 ± 15% humidity) with ad libitum access to feed and water. During a 4‐week adaptation period, mice received a semi‐synthetic diet (10% energy from fat), containing 0.14% L‐tryptophan and 30 mg NR/kg diet (Research Diet Services, Wijk bij Duurstede, The Netherlands). At 13 weeks of age, mice were stratified into 5 experimental groups on mean body weight (*n* = 12/group) and received a semi‐synthetic obesogenic diet (40% energy from fat) containing 0.14% L‐tryptophan and either 5, 15, 30, 180, or 900 mg NR per kg diet (referred to as 5NR, 15NR, 30NR, 180NR, and 900NR, respectively) for 15 weeks (diet composition in Table 1). Feed intake and body weight as well as lean and fat mass (by NMR, EchoMRI, Houston, USA) were measured weekly. In the beginning of week 16 mice were sacrificed by decapitation after 2 h of fasting. Blood was collected for immediate blood glucose measurement using a Freestyle blood glucose meter (Abbott Diabetes Care, Hoofddorp, the Netherlands). The remaining blood was centrifuged at 3000 × *g* and 4°C for 10 min and serum was stored at –80°C. Tissues were rapidly dissected, snap frozen in liquid nitrogen, and stored at –80°C (i.e. left epididymal white adipose tissue (eWAT), liver, soleus muscle, brain, mucosa scraped from small intestine). In addition, right eWAT was weighted, divided in half, and fixed for 24 h at 4°C in PBS with 4.0% formaldehyde (pH = 7.40) as described 37.

**Table 1:** Composition of diets

Ingredients (g·kg‐1 diet)	Run‐in	5NR	15NR	30NR	180NR	900NR
Casein	120.0	120.0	120.0	120.0	120.0	120.0
Wheat starch	385.9	215.7	215.7	215.7	215.7	215.7
Gelatin (hydrolyzed)	100.0	100.0	100.0	100.0	100.0	100.0
Maltodextrin	100.0	–	–	–	–	–
Sugar	100.0	100.0	100.0	100.0	100.0	100.0
Dextrose	50.0	50.0	50.0	50.0	50.0	50.0
Arbocel B800	50.0	50.0	50.0	50.0	50.0	50.0
Linseed oil	5.2	4.0	4.0	4.0	4.0	4.0
Palm oil	–	206.0	206.0	206.0	206.0	206.0
Coco oil	7.7	–	–	–	–	–
Sunflower oil	30.1	–	–	–	–	–
Mineral mixture AIN‐93	35.0	35.0	35.0	35.0	35.0	35.0
Vitamin mixture AIN‐93^a^	10.0	10.0	10.0	10.0	10.0	10.0
L‐Cystine	3.3	3.3	3.3	3.3	3.3	3.3
L‐Phenylalanine	0.4	3.5	3.5	3.5	3.5	3.5
Choline chloride 50%	2.5	2.5	2.5	2.5	2.5	2.5
Nicotinamide riboside (mg·kg‐1 diet)	30.0	5.0	15.0	30.0	180.0	900.0
Calculated amount of L‐tryptophan	1.4	1.4	1.4	1.4	1.4	1.4
Calculated energy (kJ·g‐1)	3825	4660	4660	4660	4660	4660
Energy (% of total energy content)						
Carbohydrate	66	40	40	40	40	40
Fat	10	40	40	40	40	40
Protein	23	20	20	20	20	20

a without vitamin B3

### 2.2 Indirect calorimetry

Indirect calorimetry was performed in week 14, using a PhenoMaster System (TSE Systems, Bad Homburg, Germany) as described 31. Briefly, mice were individually housed with a steady normal air flow, 12 h light–dark cycle (07:00 h lights on). Oxygen consumption, carbon dioxide production, activity (infrared beam breaks) and food and drink intake were automatically recorded. After 20 h of adaptation, ad libitum fed mice were monitored for 24 h starting from 07:00 h. Next, the mice were exposed to a fast and refeeding challenge. For this, the mice were provided with 1.5 g of fresh experimental diet at 16:00 h. The mice fully consumed this, after which they changed to a fully fasted state. The next day at 16:00 h they were provided with 1.8 g of fresh experimental diet (refeeding), which was fully consumed. Respiratory exchange ratio (RER) and energy expenditure (EE) were calculated by TSE software (TSE systems). Metabolic flexibility was assessed as described 36, with the following modifications: the maximum delta RER was calculated when switching from CHO1 (mean of 5 highest values) to FAO (mean of 5 lowest values) during fasting (maxΔRER_CHO1→FAO_), and when switching from FAO (mean of 5 lowest values) to CHO2 (mean of 5 highest values) during refeeding (maxΔRER_FAO→CHO2_).How a specific RER value relates to percentage lipid or carbohydrate oxidation can be found in 38.

### 2.3 Serum parameters

Serum triglycerides (TG) and non‐esterified fatty acids (NEFA) were measured as described 39. Serum insulin, leptin, and adiponectin were measured with a Bio‐Plex Pro Mouse Diabetes Assay using a Bio‐Plex 200 system according to manufacturer's instructions (Bio‐Rad, Veenendaal, The Netherlands). Samples were diluted 1:12.5 with sample diluent for insulin and leptin measurement and 1:1600 with serum‐based diluent for adiponectin measurement. To assess insulin resistance HOMA‐IR a was calculated as (glucose (mmol/l) × insulin (μU/ml)/22.5) 40.

### 2.4 NR metabolites in serum and liver

NR metabolites were extracted from serum as well as liver and subsequently identified and quantified using LC‐ESI‐MS/MS according to the procedure described in Supporting Methods.

### 2.5 Gene expression

Tissues were grinded in liquid nitrogen, after which RNA was isolated from liver, brain, and mucosa with a RNeasy Mini kit (Qiagen, Venlo, The Netherlands) and from eWAT with Trizol, as described 41. RNA purity and integrity was verified using Nanodrop (NanoDrop, Wilmington, USA) and Experion (Bio‐Rad, CA, USA), respectively. cDNA synthesis, followed by regular qRT‐PCR was performed as described 41. Low expressed genes were pre‐amplified for 12 cycles before qRT‐PCR using SsoAdvanced PreAmp Supermix (Bio‐Rad, CA, USA). The expression of each gene was normalized by the stably expressed reference genes using CFX Manager software (Bio‐Rad, CA, USA). Primer sequences and PCR annealing temperatures for each gene are in Supporting Information Table 1. References genes were selected based on stable expression in the selected tissues.

### 2.6 Mitochondrial density

EWAT mitochondrial DNA (mtDNA) and nuclear DNA (nDNA) were measured by qRT‐PCR as described 31. mtDNA/nDNA was calculated to assess mitochondrial density.

### 2.7 EWAT morphology

Adipocyte cell size determination as well as macrophage staining and counting were performed as described previously 37. Detailed description of these methods can be found in the Supporting methods.

### 2.8 Statistics

Data are expressed as mean ± SEM for *n* = 10‐12 mice, but *n* = 6 for IHC. Statistical analyses were performed using GraphPad Prism v5.04 (Graphpad, San Diego, CA, USA). Data were verified for normality using the D'Agostino and Pearson omnibus normality test and log transformed if needed. Body weight, cumulative feed intake, lean mass, fat mass, and mean RER were analyzed using two‐way repeated measures ANOVA (factor 1 = NR, factor 2 = WEEK) followed by Bonferroni post‐hoc analysis. All other data were analyzed using one‐way ANOVA following by Dunnett's multiple comparison test, with 30NR as control (unless otherwise stated). Pearson correlation analysis was performed on serum NR and NAD^+^, liver NR and NAD^+^ as well as leptin/adiponectin ratio and ΔRER_FAO‐CHO2_. *P*‐values < 0.05 were considered to be statistically significant.

## 3 Results

### 3.1 Body physiological parameters and metabolic flexibility

**Figure 1:**
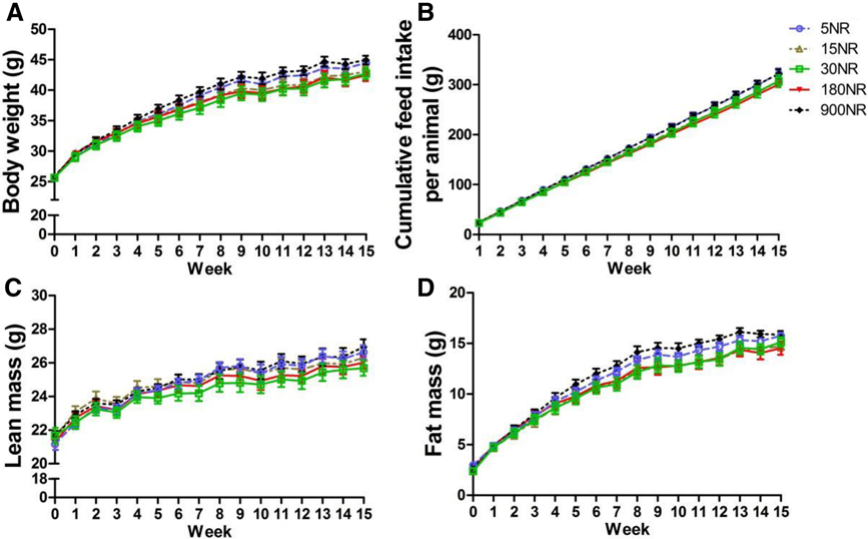
Effects of dietary NR on whole body physiological parameters. Mice were on obesogenic diets different levels of NR for 15 weeks. Body weight (A), cumulative feed intake (B), lean mass (C), fat mass (D). 5NR open circle, 15NR open upward triangle, 30NR open square, 180NR closed downward triangle, 900NR closed diamond. NR in mg/kg diet. Data are analyzed using two‐way ANOVA and presented as mean ± SEM (*n* = 11–12 mice per treatment).

**Figure 2:**
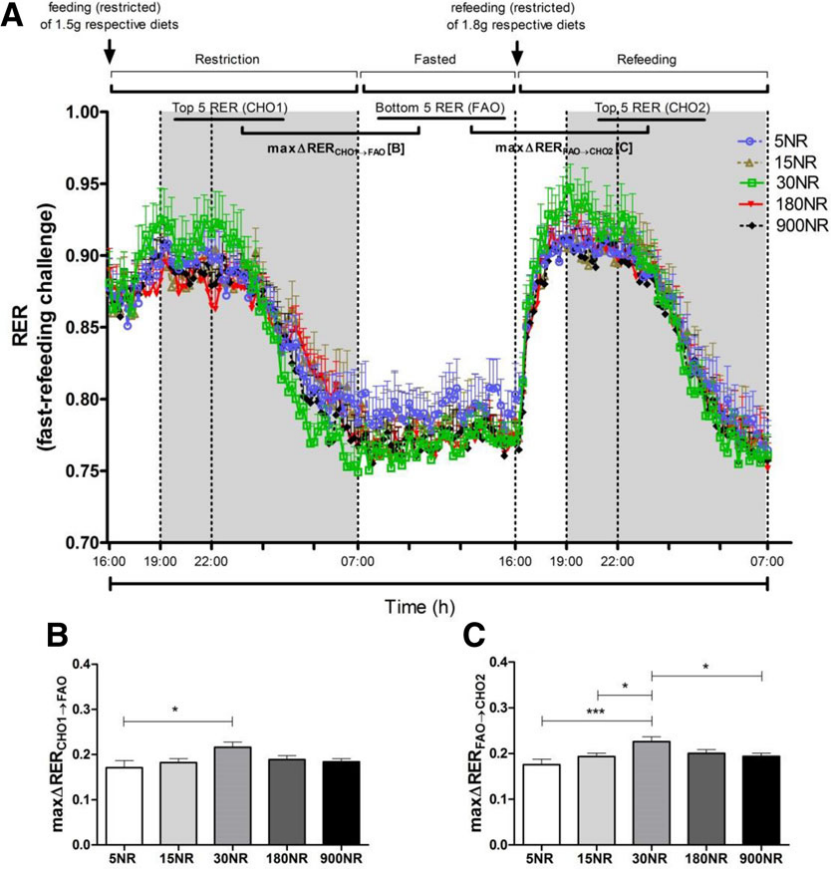
Effects of dietary NR on ΔRER during fast‐refeeding challenge at week 14. Fast‐refeeding (A): restriction (from 16:00 to 07:00 h), fasted (from 07:00 to 16:00 h), refeeding (from 16:00 to 07:00 h)periods.). 5NR open circle, 15NR open upward triangle, 30NR open square, 180NR closed downward triangle, 900NR closed diamond. Shaded areas indicate the dark, active periods. MaxΔRER_CHO1→FAO_ (B) is difference between mean of 5 highest RER values in restriction period and mean of 5 lowest RER values in fasted period. ΔRER_FAO→CHO2_ (C) is difference between mean of 5 lowest RER values in fasted period and 5 highest RER values in refeeding period. The ΔRER values represent metabolic flexibility. NR in mg/kg diet. Data are analyzed using either two‐way ANOVA (A) or one‐way ANOVA followed by Dunnett's multiple comparison test, with 30NR as control (B and C), and presented as mean ± SEM (*n* = 11–12 mice per treatment). *p<0.05, ***p<0.005.

No differences were found in body weight, cumulative feed intake, lean mass or fat mass, when comparing mice with 5, 15, 30, 180, or 900 mg NR/kg diet, after the 15‐week intervention (Fig. 1). Metabolic flexibility was assessed by calculating maxΔRER in response to a fast and refeeding in week 14 (Fig. 2A). Fasting metabolic flexibility, maxΔRER_CHO1→FAO,_ was significantly better in 30NR than in 5NR (Fig. 2B). Refeeding metabolic flexibility, maxΔRER_FAO→CHO2_, was significantly greater in 30NR than in 5NR, 15NR, or 900NR (Fig. 2C). No differences were found in energy expenditure or physical activity (Supporting Information Fig. 1). Under the non‐challenged condition no significant differences in RER or feed intake were found among NR doses (Supporting Information Fig. 2).

### 3.2 Blood glucose and serum lipids and adipokines

**Figure 3:**
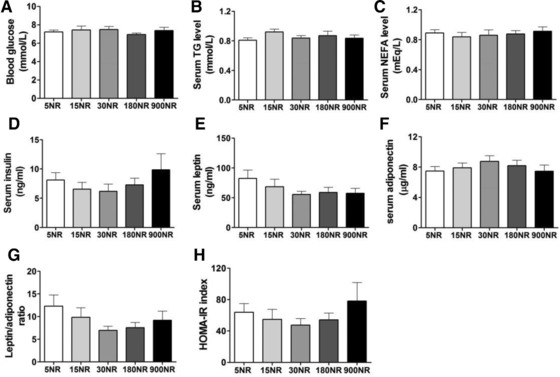
Effects of dietary NR on circulating parameters. Blood glucose (A), serum TG (B), serum NEFA (C), and serum insulin, leptin and adiponectin, respectively (D–F) after 15 weeks NR intervention. Leptin/adiponectin ratio (G) and HOMA‐IR index (H). Correlation between leptin/adiponectin ratio and metabolic flexibility (I). NR in mg/kg diet. Data are analyzed using one‐way ANOVA and presented as mean ± SEM (*n* = 11–12 mice per treatment). There is a significant difference in leptin/adiponectin ratio between 5NR and 30NR using Student's t‐test (*p* = 0.0499).

No differences were seen in blood glucose, serum TG or NEFA among NR doses (Fig. 3A–C). Serum insulin, leptin, adiponectin, leptin/adiponectin ratio, and HOMA‐IR index exhibited a tendency toward a dose‐response curve, without reaching statistical significance (Fig. 3D–H). In all cases, except adiponectin which shows opposite behavior, the measured value decreased and then increased, with 30NR being the turning point. Of note, 30NR resulted in lower leptin/adiponectin ratio compared to 5NR using *t*‐test (*p* = 0.0499), supporting our observations in metabolic flexibility.

### 3.3 Serum and liver NR metabolites

**Figure 4:**
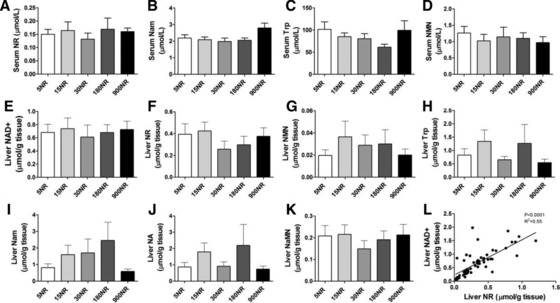
Effects of dietary NR on NR metabolites. Serum (A‐D), liver (E‐K).The correlations between NAD^+^ and NR concentration in liver (L).NR in mg/kg diet. Data are analyzed using one‐way ANOVA and presented as mean ± SEM (*n* = 10–12 mice per treatment, serum NaMN *n* = 5–10 mice).

No differences were found in levels of NR, nicotinic acid mononucleotide (NaMN), Trp or Nam (*p* = 0.0546 when comparing 900NR with 15, 30, or 180NR groups) in serum (Fig. 4A–D) and NAD^+^, NR, nicotinamide mononucleotide (NMN), Trp, Nam, NA, NaMN in liver (Fig. 4E–K) between the different groups. NAD^+^, NA, NMN could not be detected in serum, neither were the vitamin B3 status markers N‐methylnicotinamide (MeNam) and 2‐pyridone (2PY) detectable in serum.

### 3.4 eWAT morphology and gene expression

We focused the remainder of the analyses on the 5NR, 30NR, and 900NR interventions, the three most dose‐representative treatments which also showed differences in maxΔRER_FAO→CHO2_.

mRNA levels of rate limiting enzymes involved in the NR pathway, *Ido1, Ido2, Tdo2, Qprt* (de novo pathway), *Nrk1,Nrk2* (Nrk pathway) and *Nampt, Nmnat1, Nmnat3* (salvage pathway), were determined. No differences were found in the expression of these genes in either liver (Supporting Information Fig. 3A), small intestinal mucosa (Supporting Information Fig. 3B), skeletal muscle (Supporting Information Fig. 3C), or eWAT (Supporting Information Fig. 3D).

**Figure 5:**
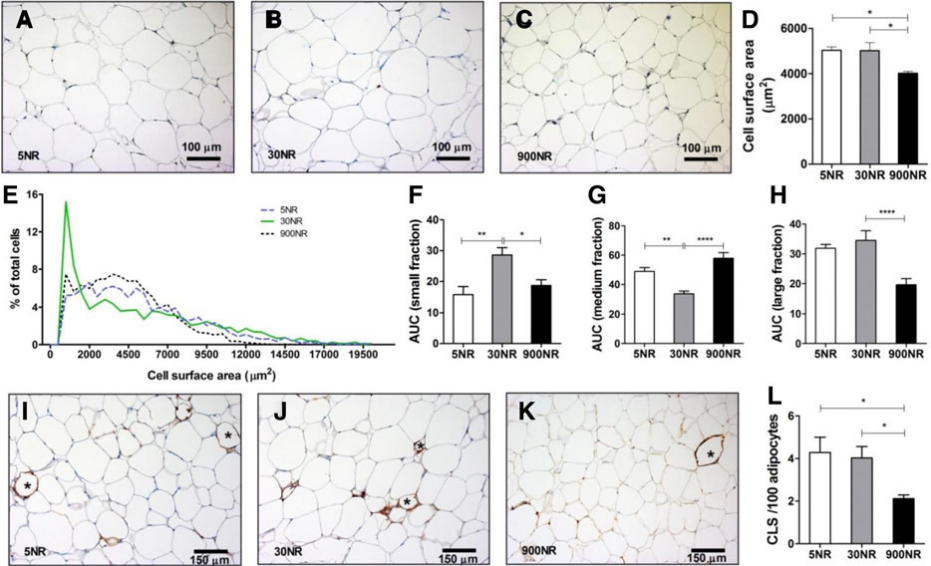
Effects of dietary NR on eWAT morphology. Representative images for 5NR (A), 30NR (B), 900NR(C); NR in mg/kg diet. Bar represents 100μm and A–C are same magnification (20×). Cell surface area (D). Frequency distribution (E) and area under the curve (AUC) of small (100–1500 μm^2^, F), medium (<1500–6000 μm^2^, Gand large (>6000μm^2^, H) fractions. Representative images of crown‐like structures (CLS, indicated by stars) for 5NR (I), 30NR (J), 900NR (K). Bar represents 150 μm and I‐K aresame magnification (20×). Total CLS number per 100 adipocytes (L). Striped 5NR, continuous 30NR, dotted 900NR. NR in mg/kg diet. Data are analyzed using one‐way ANOVA followed by Dunnett's multiple comparison test, with 30NR as control (F‐H), or Bonferroni post‐hoc test (D and L) and presented as mean ± SEM (*n* = 6 mice per treatment). *p<0.05, **p<0.01, ***p<0.005, ****p<0.001.

EWAT morphology was assessed by adipocyte size and CLS number. 900NR showed a smaller average adipocyte cell surface area (4015 ± 224.3 μm^2^) than 5NR (5032 ± 363.8 μm^2^) or 30NR (5015 ± 873.5 μm^2^) (Fig. 5A–D). Compared to the two other treatments, 30NR showed the largest number of small adipocytes (100–1500 μm^2^) as well as the smallest number of medium size adipocytes (>1500–6000 μm^2^), while 900NR showed the smallest number of large adipocytes (>6000 μm^2^) (Fig. 5E–H). 900NR showed the lowest number of CLS, compared to 5NR and 30NR (Fig. 5I–L).

**Figure 6:**
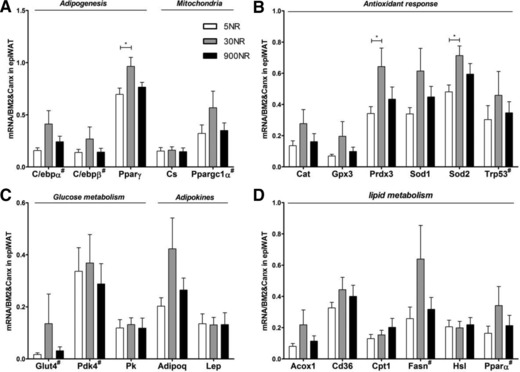
Effects of dietary NR on eWAT gene expression. mRNA levels (normalized to mean of indicated reference mRNAs) of adipogenesis and mitochondria (*C/ebpα*, *C/ebpβ, Pparγ, Cs, Ppargc1α*; A), antioxidant response (*Cat, Gpx3, Sod, Sod2, Trp53*; B), glucose metabolism and adipokine (*Glut4, Pdk4, Pk, Adipoq, Lep;* C) genes, lipid metabolism (*Acox1, Cd36, Cpt1, Fasn, Hsl, Pparα*; D). White 5NR, gray 30NR, black 900NR.^#^indicates qRT‐PCR determination was after 12‐cycle pre‐amplification. NR in mg/kg diet. Full gene names are in Supporting Information Table 2. Data are analyzed using one‐way ANOVA followed by Dunnett's multiple comparison test, with 30NR as control and presented as mean ± SEM (*n* = 10–11 mice per treatment). *p<0.05.

The expression of genes related to mitochondrial function, adipogenesis, antioxidant response, lipid metabolism, glucose metabolism as well as the adipokines leptin and adiponectin were analyzed. *Cs* was not affected by the treatments (Fig. 6A). The expression of *Pparγ*, *Sod2* and *Prdx3* was highest in 30NR, a difference that was significant compared to 5NR (Fig. 6A and B). The expression of *C/ebpα, C/ebpβ and Pgc1α*, three adipogenesis related genes involved in the regulation of *Pparγ*, showed a similar dose‐response pattern as *Pparγ*, but differences did not reach statistical significance (Fig. 6A). The expression of the anti‐oxidant genes *Cat, Gpx3, Sod1*, *Trp53* showed a dose‐response pattern similar to *Sod2* and *Prdx3*, but without reaching significance (Fig. 6B). Although we did not find differences in the expression of lipid metabolism, glucose metabolism or adipokine genes, 30NR tended to show higher levels of *Acox1, Fasn, Pparα, Glut4* and *Adipoq* (Fig. 6C and D)*. Lept* was not different, in agreement with no difference in adiposity.

## 4 Discussion

To assess beneficial and adverse effects of metabolic health, it is important that metabolic health is compromised, but not deteriorated. The mice in our study were metabolically compromised as they displayed a higher weight gain and worsened metabolic parameters compared to the same substrain of mice on an LFD in another study and comparable or slightly worse than the mice on the HFD in that study 42. On the other hand, these parameters were not fully deteriorated as, for example, in Ob/Ob mice 43. Metabolic flexibility is a biomarker for metabolic health that can be assessed non‐invasively using indirect calorimetry 31, 32. Using a fast‐refeeding challenge, we here found a dose‐response effect, showing that mice fed 30 mg NR/kg diet (30NR) were more metabolically flexible than the wide range of other NR concentrations.

By challenging homeostatic systems subtle metabolic effects, difficult to detect using static biomarkers, may be identified 44, 45. Indeed, despite an absence of changes in whole body physiological parameters and RER under the non‐challenged conditions, we did find differences in metabolic flexibility measured during a fast‐refeeding challenge. NR influenced metabolic flexibility in a dose‐response manner, with 30NR being most metabolically flexible. This was not caused by differences in activity or energy expenditure as these parameters were not influenced by NR. The dose‐dependent changes of dietary NR in metabolic flexibility might be associated with the activity of sirtuins, enzymes that are regulated by intracellular NAD+ levels and are linked to longevity and other health benefits such as improved insulin sensitivity 46, 47. The HOMA‐IR index 48 and the leptin/adiponectin ratio 49 are markers for insulin resistance. Because insulin resistance interacts with glucose and glucose disposal 29, 50, a causal relation exists. Indeed, insulin resistance and metabolic flexibility were negatively correlated in another study 51. That the HOMA‐IR index and the leptin/adiponectin ratio show a dose‐response pattern opposite to the metabolic flexibility dose‐response pattern therefore enforces our observation. In this study we used a semi‐synthetic obesogenic diet with moderate levels of fat (40 en%) resembling the average fat intake in the Netherlands and identified 30NR as the most optimal dose of NR in terms of metabolic health. Metabolic flexibility was not improved at 900NR (equivalent to 102 mg NR /kg body weigh (BW)/day at the start of the experiment to 65 mg NR/kg BW/day in the end); if anything it was decreased. This contrasts with Canto *et al*. who found that metabolic flexibility, assessed by the difference of RER between dark phase and light phase, was increased by supplementation with 400 mg NR/kg BW/day of a high fat diet that already contained 30 mg/kg diet of vitamin B3 (Nam) 8. This improvement in metabolic flexibility, on the other hand, was not observed in mice fed a high‐fat‐high‐sucrose (HFHS) diet 22, nor in mice fed a chow diet 24, even though the same high dose of NR was used. This suggests a modifying effect of the diet on improvement of metabolic flexibility at high NR concentrations. Alternatively, differences may be due to the absence and presence of the nicotinamide nucleotide transhydrogenase (*Nnt)* gene. The C57BL/6JRcc mice used in our study have, similar to humans, an intact *Nnt* gene, which is absent in the strain used by Canto *et al*. 8. NNT has a key role in redox and peroxide metabolism 52, 53 and is responsible for maintenance of mitochondrial NAD^+^ levels 54. Its absence impairs regulation of insulin secretion and thus affects glucose homeostasis 55. Absence or presence of *Nnt* thus affects disease development as well as NAD^+^ levels which may explain the observed differences in protective effects of high dose NR supplementation, which needs to be tested experimentally.

WAT plays an important role in metabolic flexibility, in addition to skeletal muscle and liver 34. Although several studies have shown a decrease in fat mass or WAT weight upon high dose NR supplementation, no WAT morphological parameters were investigated 8, 22, 56. Since adipocyte morphology and especially adipocyte size mirrors overall WAT biological function 57, we newly investigated this. 900NR decreased the average adipocyte size compared to 5NR or 30NR. However, the calculated total adipocyte number (according to Skurk T et al., 2007) 57, tended to be higher in the 900NR group as there were no differences found in eWAT weight (data not shown). 30NR showed less medium‐size adipocytes, compared to 5NR and 900NR, but had higher numbers of small (compared to 5NR and 900NR) as well as large (compared to 900NR) adipocytes. The functional differences between small and large adipocytes have been described in several studies. Smaller adipocytes tend to be more sensitive to insulin stimulation, showing a two times larger translocation of GLUT4 to the plasma membrane and increased adiponectin release 58, 59. Large adipocytes, on the other hand, have a higher ability to buffer fatty acid flux and promote lipid mobilization 60. We sacrificed the mice in the fasted state and it may have been necessary to sacrifice them at the peak of RER (3h after refeeding) to detect clear differences in the serum parameters related to adipose tissue function. Nevertheless, the adipocyte size frequency distribution in 30NR agrees with the dose‐response profile of HOMA‐IR index and serum adipokines, supporting the best metabolic flexibility at 30NR.

We examined the inflammatory state of the adipocytes by Mac‐2 staining and measuring CLS in eWAT. CLS per 100 adipocytes were lowest in 900NR compared to 5 NR and 30NR, although also there the numbers of CLS remained low. This supports the association of CLS with (clearance of) large adipocytes. Our data may also indicate that 900NR affects WAT inflammation beneficially. In agreement, treatment with 200 mg NA/kg body weight/day for 5 weeks was shown to attenuate WAT inflammation by reducing the expression of gene MCP‐1, IL‐1b and the pro‐inflammatory M1 macrophage marker CD11c in HFD‐fed mice 61. However, when taking the total adipocyte number into account, there is no statistical difference in total number of CLS in eWAT (data not shown). To definitely conclude on the effect of NR on inflammation in our study, more inflammatory markers, such as Tumor Necrosis Factor α, should be measured.

PPARγ is the master regulator of adipogenesis 62. *Pparγ* mRNA levels were significantly higher at 30NR compared to 5NR, the same trend, although not significant, was seen for *Acox1* and *Glut4*, key genes involved in fatty acid oxidation and glucose disposal. PPARγ activation in adipocytes has been shown to promote glucose metabolism by facilitating glucose uptake and enhancing glucose oxidation, as well as to increase lipid metabolism by stimulating fat uptake and mobilization and enhancing free fatty acid oxidation 63, 64. Thus, PPARγ may contribute to the increased metabolic flexibility of 30NR compared to 5NR. *Pparγ* was not further increased at 900NR. A similar pattern is seen for antioxidant defence.

SOD2 and PRDX3 were significantly elevated at the transcriptional level at 30NR compared to 5NR, suggesting a higher mitochondrial antioxidant defence activity. This was not due to a change in mitochondrial density (Supporting figure 3), confirmed by no change in expression of *Cs*. Since selective downregulation of *Sod2* and *Prdx3* in eWAT upon high fat diet feeding resulted in oxidative stress 65, their upregulation suggests increased protection against damage by reactive oxygen species (ROS). As for *Pparγ*, 900NR did not increase the expression of *Sod2* and *Prdx3 any* further. Our data suggest that adipogenesis and antioxidant response in eWAT are sensitive to low dietary NR, but not to supplemental NR.

Vitamin B3 deficiency leads to low tissue or blood NAD^+^ level and growth retardation in rodents 3, 4, 66. In our study, no decrease in body weight or body mass were observed in 5NR, neither were any vitamin B3 deficiency symptoms, such as rough skin, diarrhea seen (data are not shown). Most importantly, there were no differences between the treatments in the expression of the genes involving in NAD^+^ biosynthesis in many tissues, e.g. liver, skeletal muscle, WAT and mucosa. In line with this, NAD^+^ levels and the other NR metabolites in liver and serum did not show differ between treatments. It should be noted that the Trp content used in this study can maintain normal vitamin B3 status in mice fed a vitamin B3‐free diet 17, 18. Furthermore, NR has been shown be converted to Nam before being absorbed or reaching tissues 67, 68, where it is rapidly metabolized 69. Considering that fasted plasma Nam levels are very low 70, blood and tissue collection at the fasted status may provide another explanation for the small alteration in NR metabolites. Regretfully, we did not collect whole blood at section for the whole blood NAD measurement, which has been shown as a good biomarker for vitamin B3 status 71. Nevertheless, metabolic flexibility and gene expression in eWAT suggest that the 5NR mice were are less metabolically healthy. 5 mg NR/kg diet, with low but sufficient Trp, may therefore constitute a potential cut‐off for marginal vitamin B3 sufficiency, with metabolic flexibility as a sensitive marker. However, before this can be definitively concluded, dose‐response studies with lower vitamin B3 concentrations and other forms of vitamin B3 are needed.

In conclusion, we investigated the effects of a wide range of dietary NR on metabolic health, focusing on metabolic flexibility and WAT function. To the best of our knowledge, this is the first time that NR is used as an exclusive source of vitamin B3, applied in a range between marginal sufficiency and supplemental amounts in the context of an mildly obesogenic diet in a rodent study. Based on metabolic flexibility, the data linked to WAT function and no effect on growth, we conclude that 30 mg NR/kg diet constitutes the optimal concentration to support metabolic health.

*The authors have declared no conflict of interest*.

## Supporting information


**Supporting table 1** *Sequences of primers for qRT‐PCR*
**Supporting Figure 1**. *Effects of dietary NR on energy expenditure and activity.A*ctivity (A) and energy expenditure (B) during the fast and refeeding challenge were measured in indirect calorimetry in week 14.5NR white, 15NR light grey, 30NR grey, 180NR dark grey, 900NR black.NR in mg/kg diet. Data are analyzed using one‐way ANOVA and shown as mean ± SEM (n=11‐12 mice per treatment).
**Supporting figure 2**.*Effects of dietary NR on RER during Indirect Calorimetry*.RER under ad libitum conditions was measured for 24hrs prior to the fast and refeeding challenge in indirect calorimetry in week 14. ). 5NR open circle, 15NR open upward triangle, 30NR open square, 180NR closed downward triangle, 900NR closed diamond. NR in mg/kg diet. NR in mg/kg diet: Shaded areas indicated dark, active periods. Data are analyzed using two‐way ANOVA and shown as mean ± SEM (n=11‐12 mice per treatment).
**Supporting Figure 3**.*Effects of dietary NR on expression of NR metabolism genes*.mRNA levels (normalized to mean of indicated reference mRNAs) of de novo pathway (*Tdo2, Ido1, Ido2, Qprt*), Nrk pathway (*Nrk1, Nrk2*), salvage pathway (*Nampt, Nmnat1, Nmnat3*) in liver (A), small intestinal mucosa (B), *soleus* muscle (C), eWAT (D). White 5NR, grey 30NR, black 900NR.^#^ indicates qRT‐PCR determination was after 12‐cycle pre‐amplification. Full gene names are in Supporting Table 2. Data are analyzed using one‐way ANOVA and shown as mean ± SEM (n=11‐12 mice per treatment).
**Supporting Figure 4**.*Mitochondrial density in epiWAT*.Ratio of mitochondrial over nuclear DNA.White 5NR, grey 30NR, black 900 NR.NR in mg/kg diet. Data are analyzed using one‐way ANOVA and shown as mean ± SEM (n=11‐12 mice per treatment).
