# Supplementary material for: Effects of a wide range of dietary nicotinamide riboside (NR) concentrations on metabolic flexibility and white adipose tissue (WAT) of mice fed a mildly obesogenic diet
Source: Mol Nutr Food Res. 2017 Apr 13;61(8):1600878. doi: 10.1002/mnfr.201600878 (PMC5573990; doi:10.1002/mnfr.201600878)
Supplement: Supplementary file 1 — Supporting table 1 Sequences of primers for qRT‐PCR Supporting Figure 1. Effects of dietary NR on energy expenditure and activity.Activity (A) and energy expenditure (B) during the fast and refeeding challenge were measured in indirect calorimetry in week 14.5NR white, 15NR light grey, 30NR grey, 180NR dark grey, 900NR black.NR in mg/kg diet. Data are analyzed using one‐way ANOVA and shown as mean ± SEM (n=11‐12 mice per treatment). Supporting figure 2.Effects of dietary NR on RER during Indirect Calorimetry.RER under ad libitum conditions was measured for 24hrs prior to the fast and refeeding challenge in indirect calorimetry in week 14. ). 5NR open circle, 15NR open upward triangle, 30NR open square, 180NR closed downward triangle, 900NR closed diamond. NR in mg/kg diet. NR in mg/kg diet: Shaded areas indicated dark, active periods. Data are analyzed using two‐way ANOVA and shown as mean ± SEM (n=11‐12 mice per treatment). Supporting Figure 3.Effects of dietary NR on expression of NR metabolism genes.mRNA levels (normalized to mean of indicated reference mRNAs) of de novo pathway (Tdo2, Ido1, Ido2, Qprt), Nrk pathway (Nrk1, Nrk2), salvage pathway (Nampt, Nmnat1, Nmnat3) in liver (A), small intestinal mucosa (B), soleus muscle (C), eWAT (D). White 5NR, grey 30NR, black 900NR.# indicates qRT‐PCR determination was after 12‐cycle pre‐amplification. Full gene names are in Supporting Table 2. Data are analyzed using one‐way ANOVA and shown as mean ± SEM (n=11‐12 mice per treatment). Supporting Figure 4.Mitochondrial density in epiWAT.Ratio of mitochondrial over nuclear DNA.White 5NR, grey 30NR, black 900 NR.NR in mg/kg diet. Data are analyzed using one‐way ANOVA and shown as mean ± SEM (n=11‐12 mice per treatment). [file MNFR-61-na-s001.docx]

**Supporting information**

Effects of a wide range of dietary nicotinamide riboside (NR) concentrations on metabolic flexibility and white adipose tissue (WAT) of mice fed a mildly obesogenic diet.

Wenbiao Shi, Maria A. Hegeman, Dorien A.M. van Dartel, Jing Tang, Manuel Suarez, Hans Swarts, Bart van der Hee, Lluis Arola and Jaap Keijer

**Supporting methods**

*NR metabolites in serum and liver*

Metabolite extraction from serum was performed as described before [1]. Briefly, 100 µL serum was vortexed with 200 µL acetonitrile (ACN) for 90 seconds, kept for 20 minutes at 4ºC and centrifuged for 10 minutes at 16,600*g*, 4ºC. 150 µL supernatant was evaporated under nitrogen flow until samples were dry.

Liver extraction was done as described previously [2] with the following modifications: 30 mg lyophilized tissue was vortexed in 0.5mL physiological saline for 30 seconds, followed by 30 seconds sonication (Vibra Cell, Sonics, Newton, USA). 0.5 mL acetone was added, vortexed and centrifuged for 15 minutes at 10,000*g*, 4ºC. This procedure was repeated three time, after which all 3 upper phases were combined, and evaporated under nitrogen flow. Dried serum or liver samples were dissolved in 200 µL ACN:milliQ water (50:50, v/v) filtered through 0.45 µm, respectively. 10 µL. BP3 at 25 ppm for serum or 20 µL for liver samples was added prior to extraction as internal standard.

NR metabolites were identified and quantified using an LC-ESI-MS/MS system consisting of an Agilent HPLC 1200 Series coupled to a triple quadrupole mass spectrometer 6410 (both Agilent Technologies, CA, U.S.A.). Chromatographic separation was done at 30ºC with a flow rate at 0.6 mL/minute using a XBridge Amide 3.5 µm; 100 mm x 2.1 mm i.d. column (Waters, Hertfordshire, U.K.). Solvent A was 10 mM ammonium formiate 0.1% formic acid and solvent B was acetonitrile. Separation was done using the following gradient: initially phase B was set at 90% and it was reduced gradually up to 50% B in 15 minutes. Then it was maintained isocratically for 5 minutes and finally it was returned to initial conditions for 2 minutes. A post-run of 6 minutes was set between samples to re-equilibrate the column. Analyses were performed in the positive mode in electrospray ionization (ESI), with a drying gas temperature of 325ºC and flow rate of 9 L/minute. Nebulizer gas pressure was 30 psi and the capillary voltage was set at 4000V. The selected reaction monitoring transitions (SRM) and individual fragmentor voltage and collision energy for each compound were evaluated using commercial standards to obtain the best instrumental conditions. Two transitions were acquired for each compound, one for quantification and a second for confirmation purposes. The selected SRM transitions were 255<123 and 255<255 for NR, 124<53 and 124<80 for NA, 123<53 and 123<80 for Nam, 205<146 and 205<188 for Trp, 335<123 and 335<97 for nicotinamide mononucleotide (NMN), 664<427 and 664<523 for NAD and 336<124 and 336<97 for nicotinic acid mononucleotide (NaMN), 96<78 and 96<51 for 2-pyridone (2PY) and 137<108 and 137<80 for N-methylnicotinamide (MeNam). Data acquisition and treatment was carried out using Masshunter software.

*EWAT morphology*

Adipocyte size as well as macrophage staining and counting were by immunohistochemistry (IHC) as described [3]. Briefly, tissue was fixed, washed in PBS, embedded in paraffin and sectioned at 5 µm using an automated microtome (Microm GmbH, Heidelberg, Germany). Sections after 20 sequential cuts were used to ensure no repetitive adipocytes were present. Tissue sections were deparaffinised, rehydrated and then stained with Mayer’s haematoxylin for 30 seconds (Vector, CA, U.S.A.). Representative pictures were photographed and adipocyte size was measured using Axiovision (Zeiss, Munich, Germany) and expressed in surface area (μm^2^) per adipocyte. The frequency distribution of adipocyte size was calculated as described [4], with some modifications. Briefly, adipocyte surface area was distributed in 100 μm^2^ clusters in Excel and subsequently clustered as defined fractions of small (100-1500 μm^2^), medium (>1500-6000 μm^2^), and large (>6000 μm^2^). Values less than 100 μm^2^ were excluded. Crown-like structures (CLS) from 1000 adipocytes per animal were counted, and expressed as CLS/100 adipocytes.

**Supporting table 1**

*Sequences of primers for qRT-PCR*

| gene | primer forward 5’-3’ | | primer reverse 5’-3’ | AT  °Ϲ |
| --- | --- | --- | --- | --- |
| Acox1 | | TGCGGTGGGCACGGCTATTC | CGCTGGCTCGGCAGGTCATT | 60 |
| Actb (β-actin)# | | GGGATGTTTGCTCCAACCAA | GCGCTTTTGACTCAAGGATTTAA | 60 |
| Adipoq | | CCCATGAGTACCAGACTAATGAGACC | TGACTGGGCAGGATTAAGAGGAAC | 60 |
| B2m# | | CCCCACTGAGACTGATACATACGC | AGAAACTGGATTTGTAATTAAGCAGGTTC | 60 |
| Canx# | | GCAGCGACCTATGATTGACAACC | GCTCCAAACCAATAGCACTGAAAGG | 60 |
| Cat | | CTCGCAGAGACCTGATGTCC | TGTGGAGAATCGAACGGCAA | 60 |
| Cd36 | | GACGCAGCCTCCTTTCC | GGCATTGGCTGGAAGAAC | 60 |
| Cebpa (C/Ebpα) | | GCCAAACTGAGACTCTTCACTAACG | CACTACTACATACACCCTTGGACAAC | 60 |
| Cebpb(C/EBPβ) | | GAGCGACGAGTACAAGATGCG | GCTGCTCCACCTTCTTCTGC | 60 |
| Cpt1 | | CTGAGACAGACTCACACCGC | GTGGAGCCTACGGTTGTTCT | 58 |
| Cs | | ACAGTGAAAGCAACTTCGCC | GTCAATGGCTCCGATACTGC | 58 |
| Fasn | | GTGCAGAGCTGTGCTCCTGA | GTGCAGAGCTGTGCTCCTGA | 55 |
| Gpx3 | | CCATTCGGCCTGGTCATTCT | GGAGGGCAGGAGTTCTTCAG | 60 |
| Hprt1# | | TGACACTGGTAAAACAATGCAAACTTTG | GAGGTCCTTTTCACCAGCAAGCT | 60 |
| Hsl | | TCAGGGACAGAGGCAGAGGAC | TCCACTTAGTTCCAGGAAGGAGTTG | 58 |
| Ido1 | | TCTGCTGTATGAGGGGGTCT | GGAGATTCTTTGCCAGCCTC | 60 |
| Ido2 | | ATTGCCCTCAGACTTCCTCAC | TCTTGGCAGCACCTTTTGGG | 60 |
| Lep | | GGCTTTGGTCCTATCTGTCTTATGTTC | CCCTCTGCTTGGCGGATACC | 60 |
| Nuclear DNA | | CTTAGAGGGACAAGTGGCGTTC | CGCTGAGCCAGTCAGTGTAG | 58 |
| Mito DNA | | CCGCAAGGGAAAGATGAAAGAC | TCGTTTGGTTTCGGGGTTTC | 60 |
| Nampt | | GATTGAGACTATTCTTGTTCAGT | GTAACTTGTATTCCAGACCATC | 60 |
| Nmnat1 | | CCAAACCAACAGGTGTGCC | CCACGATTTGCGTGATGTCC | 60 |
| Nmnat3 | | TAGCCCCACGGTCACTTTTC | GCAGTGGCCACCCTGTTTTA | 60 |
| Nmrk1 | | CTTGAAGCTTGCTCTGCGAC | CTCCGTTTGTCACACCACCA | 60 |
| Nmrk2 | | CGGGGTGGAAGTGGTCTATTT | GGACCATACAGGACGCCAG | 60 |
| Pdk4 | | TCAGTGACTCAAAGACGGGAAACC | TGTGGTGAAGGTGTGAAGGAACG | 60 |
| Pk | | CAAGTCTGGCAGGAGTGC | TTCAGCACGGCATCCTTA | 60 |
| Ppara (Pparα) | | AAGAACCGGAACAAATGCCAGTAC | TCTTCAGGTAGGCTTCGTGGATTC | 59 |
| Pparg (Pparγ) | | GAAGTTCAATGCACTGGAATTAGATGAC | TTGTCTTGGATGTCCTCGATGGG | 60 |
| Ppargc1a (Pgc1α) | | CCCTGCCATTGTTAAGACC | TGCTGCTGTTCCTGTTTTC | 60 |
| Prdx3 | | GTGGTTTGGGCCACATGAAC | AGAGACCTCTGAGCGCAATG | 60 |
| Qprt | | AGACAACCATGTAGTGGCGG | TGCAGCTCCTCAGGCTTAAA | 60 |
| Rpl4# | | ACAACAGACAGCCCTATGCC | CCCCCACGACACATATTTCCA | 58 |
| Rps15# | | CGGAGATGGTGGGTAGCATGG | ACGGGTTTGTAGGTGATGGAGAAC | 60 |
| Slc2a4 (Glut4) | | CCATTCCCTGGTTCATTGTG | GTTTTGCCCCTCAGTCATTC | 60 |
| Sod1 | | TCGGCTTCTCGTCTTGCTCTC | GTTCACCGCTTGCCTTCTGC | 60 |
| Sod2 | | TTCTGGACAAACCTGAGCCCTAAG | GCAGCAATCTGTAAGCGACCTTG | 60 |
| Tdo2 | | ACTGTGAGCGACAGGTACAA | CTGTCACTGTACTCGGCTGT | 60 |
| Trp53 | | AGTATTTCACCCTCAAGATCCGC | AGCAGTTTGGGCTTTCCTCC | 60 |

AT=annealing temperature

# = reference genes

*Acox1*, Acyl-coenzyme A oxidase 1; *Actb*, Actin beta; Adipoq, Adiponectin; *B2m,* Beta-2-Microglobulin; *Canx*, Calnexin; *Cat*, Catalase; *Cd36*, Cluster of differentiation 36; *C/ebpα*, CCAAT/enhancer binding protein alpha; *C/ebpβ*, CCAAT/enhancer binding protein beta; *Cpt1*, Carnitine palmitoyltransferase-1; *Cs*, Citrate synthase; *Fasn*, Fatty acid synthase; *Gpx3*, Glutathione peroxidase 3; *Hprt1*, Hypoxanthine Phosphoribosyltransferase 1; *Hsl*, Hormone-sensitive lipase; *Ido1*, Indoleamine 2,3-dioxygenase 1; *Ido2*, Indoleamine 2,3-dioxygenase 2; *Lep*, Leptin; *Nuclear DNA; Mito DNA*, Mitochondrial DNA; *Nampt*, Nicotinamide phosphoribosyltransferase; *Nmnat1*, Nicotinamide mononucleotide adenylyltransferase 1; *Nmnat3*, Nicotinamide mononucleotide adenylyltransferase 3; *Nrk1*, Nicotinamide riboside kinase 1; *Nrk2*, Nicotinamide riboside kinase 2; *Pdk4*, Pyruvate dehydrogenase kinase 4; *Pk*, Pyruvate kinase; *Pparα*, Peroxisome proliferator-activated receptor alpha; *Pparγ*, Peroxisome proliferator-activated receptor gamma; *Ppargc1α*,Peroxisome proliferator-activated receptor gamma coactivator 1-alpha; *Prdx3*, Peroxiredoxin 3; *Qprt*, Quinolinate phosphoribosyltransferase; *Rpl4*, Ribosomal Protein L4; *Rps15*, Ribosomal Protein S15; *Slc2a4*, Facilitated glucose transporter 4; *Sod1*, Superoxide dismutase 1; *Sod2*, Superoxide dismutase 2; *Tdo2*, Tryptophan 2,3-dioxygenase; *Trp53*, Transformation related protein 53.

**Supporting Figure**

**Supporting Figure 1.** *Effects of dietary NR on energy expenditure and activity. A*ctivity (A) and energy expenditure (B) during the fast and refeeding challenge were measured in indirect calorimetry in week 14. 5NR white, 15NR light grey, 30NR grey, 180NR dark grey, 900NR black. NR in mg/kg diet. Data are analysed using one-way ANOVA and shown as mean ± SEM (n=11-12 mice per treatment).


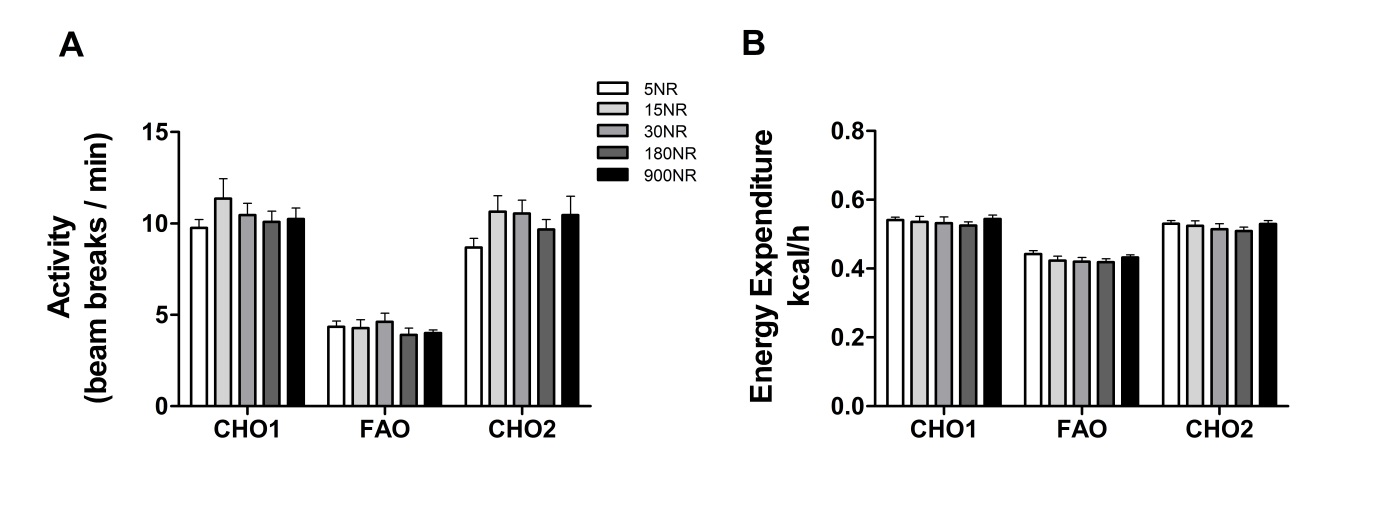


**Supporting figure 2.** *Effects of dietary NR on RER* *during Indirect Calorimetry.* RER under *ad libitum* conditions was measured for 24hrs prior to the fast and refeeding challenge in indirect calorimetry in week 14. ). 5NR open circle, 15NR open upward triangle, 30NR open square, 180NR closed downward triangle, 900NR closed diamond. NR in mg/kg diet. NR in mg/kg diet: Shaded areas indicated dark, active periods. Data are analysed using two-way ANOVA and shown as mean ± SEM (n=11-12 mice per treatment).


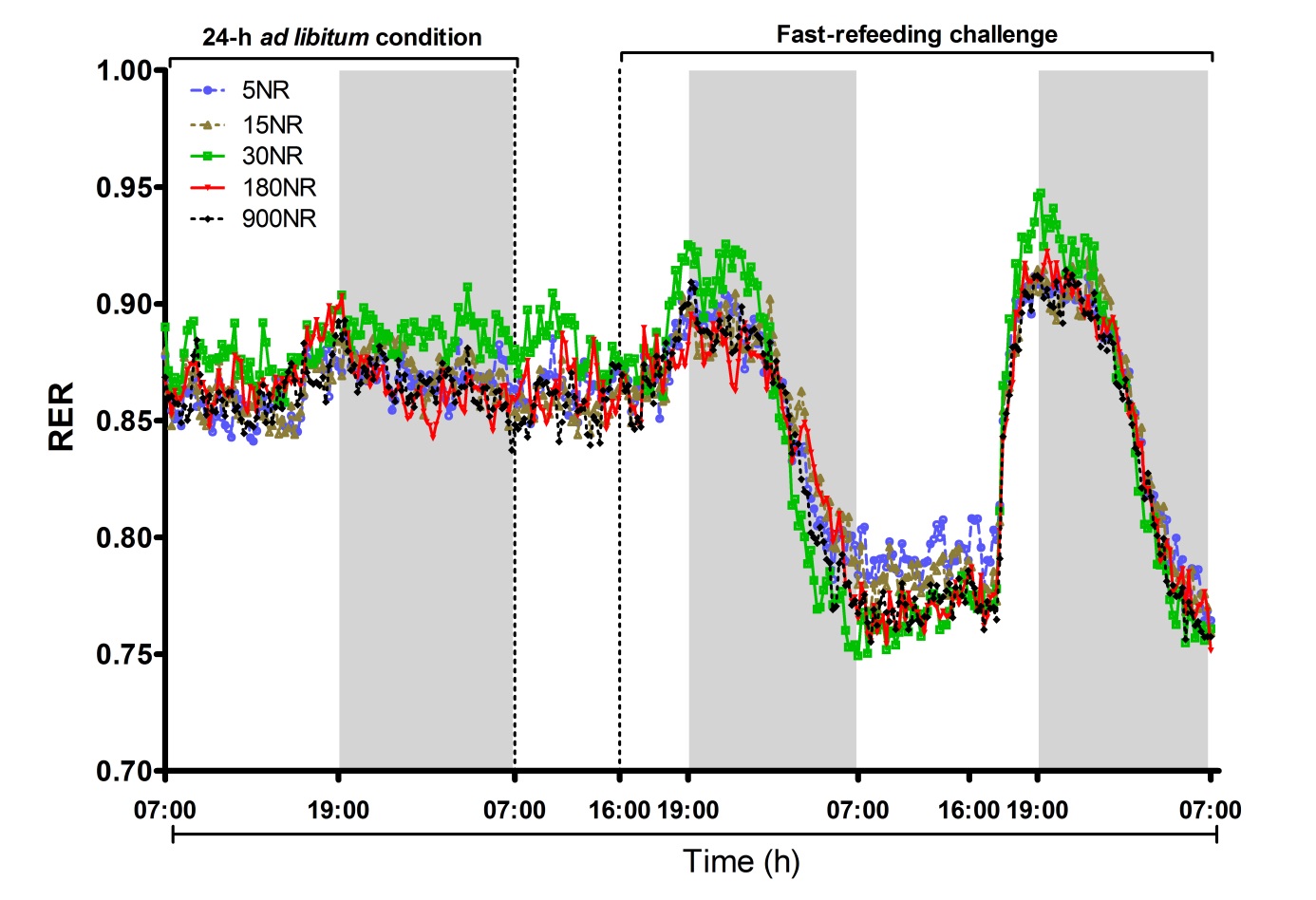


**Supporting Figure 3.** *Effects of dietary NR on expression of NR metabolism genes.* mRNA levels (normalized to mean of indicated reference mRNAs) of de *novo* pathway (*Tdo2, Ido1, Ido2, Qprt*), Nrk pathway (*Nrk1, Nrk2*), salvage pathway (*Nampt, Nmnat1, Nmnat3*) in liver (A), small intestinal mucosa (B), *soleus* muscle (C), eWAT (D). White 5NR, grey 30NR, black 900NR. ^#^ indicates qRT-PCR determination was after 12-cycle pre-amplification. Full gene names are in Supporting Table 2. Data are analysed using one-way ANOVA and shown as mean ± SEM (n=11-12 mice per treatment).


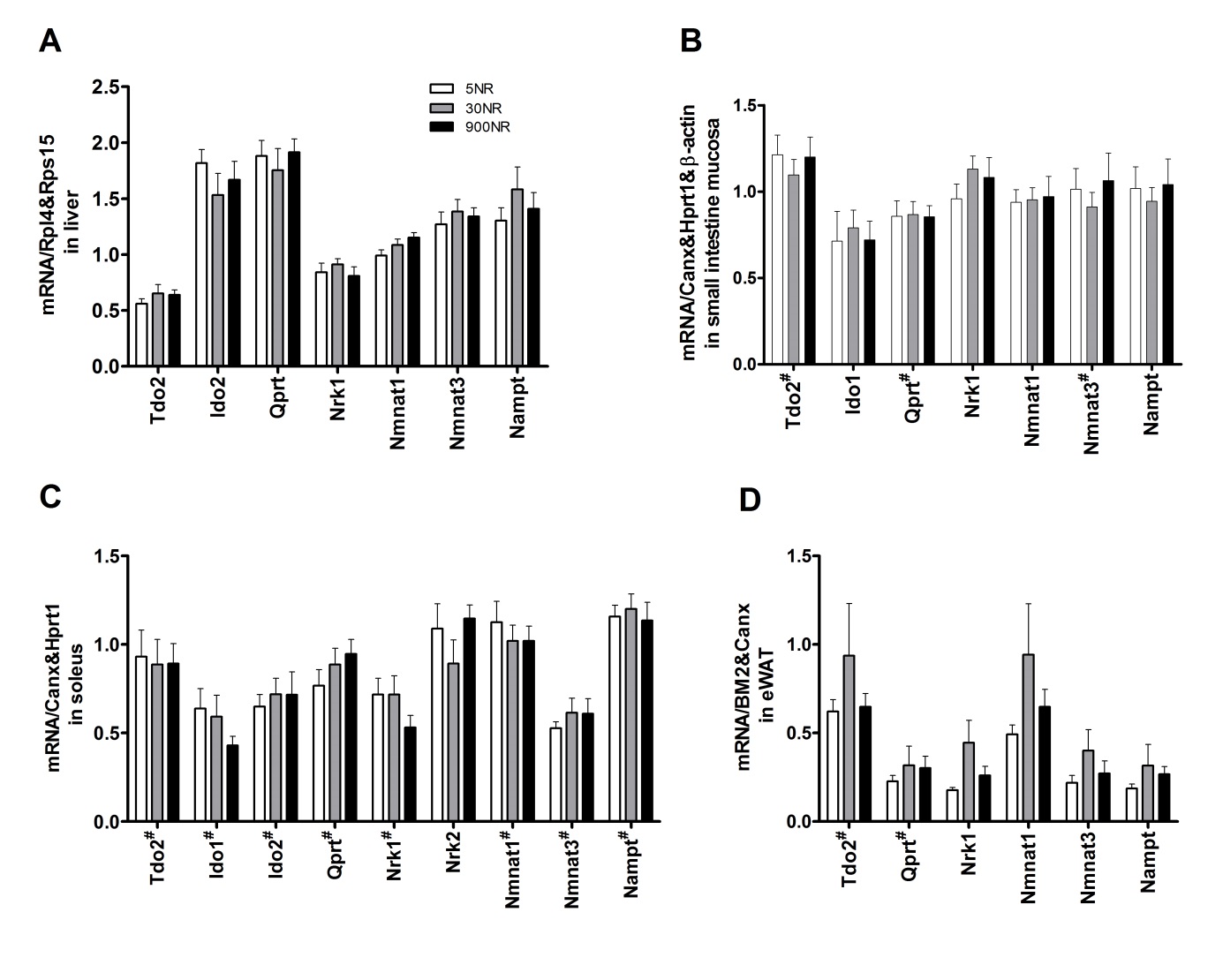


**Supporting Figure 4.** *Mitochondrial density in epiWAT.* Ratio of mitochondrial over nuclear DNA. White 5NR, grey 30NR, black 900 NR. NR in mg/kg diet. Data are analysed using one-way ANOVA and shown as mean ± SEM (n=11-12 mice per treatment).


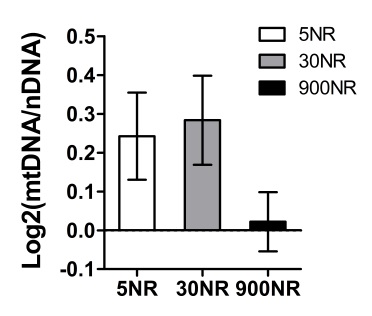


***Supporting references***

[1] Szafarz, M., Lomnicka, M., Sternak, M., Chlopicki, S., Szymura-Oleksiak, J., Simultaneous determination of nicotinic acid and its four metabolites in rat plasma using high performance liquid chromatography with tandem mass spectrometric detection (LC/MS/MS). *J. Chromatogr. B* 2010, *878*, 895-902.

[2] Aragones, G., Suarez, M., Ardid-Ruiz, A., Vinaixa, M.*, et al.*, Dietary proanthocyanidins boost hepatic NAD(+) metabolism and SIRT1 expression and activity in a dose-dependent manner in healthy rats. *Sci. Rep.* 2016, *6*, 24977.

[3] Hoek-van den Hil, E. F., van Schothorst, E. M., van der Stelt, I., Swarts, H. J.*, et al.*, Direct comparison of metabolic health effects of the flavonoids quercetin, hesperetin, epicatechin, apigenin and anthocyanins in high-fat-diet-fed mice. *Genes Nutr.* 2015, *10*, 469.

[4] Parlee, S. D., Lentz, S. I., Mori, H., MacDougald, O. A., Quantifying size and number of adipocytes in adipose tissue. *Methods Enzymol.* 2014, *537*, 93-122.
